# Prediction model for suicide based on back propagation neural network and multilayer perceptron

**DOI:** 10.3389/fninf.2022.961588

**Published:** 2022-08-11

**Authors:** Juncheng Lyu, Hong Shi, Jie Zhang, Jill Norvilitis

**Affiliations:** ^1^School of Public Health, Weifang Medical University, Weifang, China; ^2^Shandong Ikang Group, Weifang Ikang Guobin Medical Examination Center, Weifang, China; ^3^Department of Sociology, Central University of Finance Economics, Beijing, China; ^4^Department of Sociology, State University of New York Buffalo State, Buffalo, NY, United States

**Keywords:** suicide, BP neural network, multilayer perceptron, prediction model, China

## Abstract

**Introduction:**

The aim was to explore the neural network prediction model for suicide based on back propagation (BP) and multilayer perceptron, in order to establish the popular, non-invasive, brief and more precise prediction model of suicide.

**Materials and method:**

Data were collected by psychological autopsy (PA) in 16 rural counties from three provinces in China. The questionnaire was designed to investigate factors for suicide. Univariate statistical methods were used to preliminary filter factors, and BP neural network and multilayer perceptron were employed to establish the prediction model of suicide.

**Results:**

The overall percentage correct of samples was 80.9% in logistic regression model. The total coincidence rate for all samples was 82.9% and the area under ROC curve was about 82.0% in the Back Propagation Neural Network (BPNN) prediction model. The AUC of the optimal multilayer perceptron prediction model was above 90% in multilayer perceptron model. The discrimination efficiency of the multilayer perceptron model was superior to BPNN model.

**Conclusions:**

The neural network prediction models have greater accuracy than traditional methods. The multilayer perceptron is the best prediction model of suicide. The neural network prediction model has significance for clinical diagnosis and developing an artificial intelligence (AI) auxiliary clinical system.

## Introduction

While world suicide rates are slightly on the rise (WHO, 2014), China is experiencing a tremendous decline in its suicide rate in recent decades (Jiang et al., 2018). Although the Chinese suicide rate has decreased recently, it is still a leading cause of death (Zhang et al., 2011a; Liu et al., 2017) and serious threat to health in China. The average suicide rate in China was 6.75/100,000 from 2012 to 2015 years (Liu et al., 2017). This represents a decrease of about 65% (with national rate of 7.21/100,000) in China (Jiang et al., 2018), although this varies by gender, age, and location (Chen et al., 2019).

To date, there have been several studies examining risk factors for suicide in China. Phillips et al. (2002) researched the risk factors for suicide in China through a national case-control psychological autopsy (PA) study. Sun et al. (2011) report an annual peak in suicide in the spring and early summer. Scholars have explored sociological reasons for the trend of declining suicide rate in China and found that the declining of psychological strain was the main reasons (Zhang et al., 2011a). Further, risk factors, coping skills, and mental disorders among rural youth suicide in China have been examined in recent years (Zhang et al., 2011b; Li and Zhang, 2012). Subjects at risk of suicidal behavior usually search the information of self-harm and suicidal behaviors on internet, particularly for the young adolescents. The relationship between suicide-rates and suicide-related search volumes by internet still have been reported (Solano et al., 2016).

Much of the previous literature has focused on risk factors and established prediction models using traditional statistical methods. However, because the suicide population is unique, the distribution of the variables is likely to be skewed and the relationships are likely to non-linear (Lyu and Zhang, 2019). But most previous literature has examined prediction models through logistic regression (Lyu and Zhang, 2019), which is not suitable for the data of special populations. However, the neural network model is more robust to identify complex non-linear relationships between variables.

Artificial Neural Network (ANN) has been applied in medical research fields (Jiang and Jianfeng, 2011; Marques et al., 2020; Chiang et al., 2021; Hooper et al., 2021; Yang and Yu, 2021). There has been little literature applying the neural network model to establish the predicted model to discriminate patient or not (Modai et al., 2002). As the amount of data continues to grow at an almost incomprehensible rate, machine learning algorithms is better suitable to the present-day era of big data and data science. There are many famous algorithms such as Neural Network, Logistic Regression, SVM, Multilayer Perceptron, Naive Bayes, K-Means, Random Forest, etc. Back propagation (BP) neural network and multilayer perceptron are two classic artificial intelligence (AI) machine learning methods. They have been widely used in engineering and scientific research fields. With the development of AI and computer technology, there is a few of research have tried to apply the machine learning method to suicide and suicide attempt (Mezuk et al., 2018; Ryu et al., 2019; Chen et al., 2020; Gradus et al., 2020; Boudreaux et al., 2021). But there are few studies establishing the prediction model for suicide using the ANN. Furthermore, in much of the existing research, the predictor variables are difficult to measure or are not common in the general population. So it is important to develop a brief, easily administered, and widely applicable model for predicting suicide risk using AI methods basing on BP neural network and multilayer perceptron.

The current study aimed to establish the prediction model of suicide through logistic regression, Back Propagation Neural Network (BPNN) and multilayer perceptron methods, respectively, in order to verify the application of the neural network model and compare the accuracy of the discriminant effects. Because ANN can handle the complex fuzzy mapping relations and identify complex non-linear relationships between variables by simulating the human brain intelligently, so the research hypothesis was that the neural network model would be more accurate than the traditional method.

## Materials and methods

### Data collection

Data came from a large case-control designed survey carried out in 16 counties (Xian) from three provinces (Shandong, Liaoning and Hunan provinces) in China. Shandong, Liaoning and Hunan province is locate in north, northeast, and south in China, respectively. And the countries (*Xian*) were chosen by the random sample method from each province. In China, all death incidents are strictly required to be reported to the civil affairs agency in order to obtain death certificates. Village doctors were recruited to collect the death certificate and report the death to the Centre for Disease Control (CDC). The suicide cases were traced by the death reporting system of CDC. In order to control the selective bias, strict inclusion and exclusion criteria were established. After suicide cases were identified, cases were confirmed and verified by the experts in order to exclude cases of deliberate self-injury, accidental injury, mistaken ingestion of drugs or pesticides (Lyu et al., 2018). After the suicide cases were confirmed, a living community control, matched for gender, community and age (± 2 years), was randomly selected for each suicide case.

Data for each suicide case and control were collected by the PA method. In order to decrease biased responses, two informants for each case and control were enlisted. The first informant was always the parent, spouse or another family member, and the second informant was always a close friend, co-worker or neighbor. The informants were interviewed by the professionally trained interviewer. After interviews, the responses from the two informants were combined using the principles of information integration (Fang, 2009). A detailed description of the data collection can be reviewed in a prior publication (Lyu et al., 2016).

### Measurements

The questionnaires were self-designed to collect data, which main included demographic variables, the predictor variables or influencing factors and the widely used psychological scales. The information of the questionnaires is as followings.

Demographic variables such as age, gender, residence, highest degree earned, education year, marital status, whether or not lived alone were measured. The survey items, response options and recoding methods are shown in Table 1.

**TABLE 1:** The demographic variables of suicide case and control groups.

Variables	Options and record	Control (*n* = 416)	Case (*n* = 392)	χ^2^/t/*t’*	*P*
Age	Full year	25.69 ± 6.16	26.84 ± 6.37	–2.591	0.010
Gender	Male = 0	202 (48.6%)	214 (54.6%)	2.942	0.086
	Female = 1	214 (51.4%)	178 (45.4%)		
Residence	Urban = 1	19 (4.6%)	10 (2.6%)	2.371	0.124
	Rural = 2	397 (95.4%)	382 (97.4%)		
Highest degree	Elementary = 1	63(15.2%)	165 (42.9%)	85.628	< 0.001
	Middle school = 2	251 (60.5%)	182 (47.3%)		
	High school = 3	74 (17.8%)	32 (8.3%)		
	College or above = 4	27 (6.5%)	6 (1.6%)		
Education years	Years	9.15 ± 2.40	7.38 ± 2.77	9.605	< 0.001^#^
Marital status	Single/Never married = 0	145 (34.9%)	168 (42.9%)	5.445	0.020
	Ever married = 1	271 (65.1%)	224 (57.1%)		
Live alone	No = 0	399 (95.9%)	357 (91.1%)	7.858	0.005
	Yes = 1	17 (4.1%)	35 (8.9%)		

^#^Indicated the adjusted t-test.

The possible predictors of suicide such as number of family members, spousal relation, relation with parents, status in family, family financial status, superstition, health condition, severe chronic disease, mental illness, family mental disorder history, family suicide history, and pesticide stored at home, as well as whether aspirations were reached or not were assessed through the self-designed questionnaire. The options and records of the items are shown in Table 2.

**TABLE 2:** Predictors of suicide in case and control groups.

Variables	Options and record	Control (n = 416)	Case (n = 392)	χ^2^/t	*P*
No. of family Members	Number	4.08 ± 1.19	3.81 ± 1.43	2.989	0.003
Spousal Relation	Excellent = 1	62 (23.3%)	13 (5.8%)	120.729	< 0.001
	Good = 2	176 (66.2%)	95 (42.2%)		
	Average = 3	28 (10.5%)	64 (28.4%)		
	Not good = 4	0 (0.0%)	28 (12.4%)		
	Poor = 5	0 (0.0%)	25 (11.1%)		
Relation with Parents	Excellent = 1	129 (31.4%)	43 (11.2%)	121.484	< 0.001*
	Good = 2	244 (59.4%)	193 (50.1%)		
	Average = 3	38 (9.2%)	113 (29.4%)		
	Not good = 4	0 (0.0%)	27 (7.0%)		
	Poor = 5	0 (0.0%)	9 (2.3%)		
Status in family	Highest = 1	21 (5.1%)	30 (7.7%)	64.802	< 0.001
	High = 2	227 (54.7%)	132 (33.8%)		
	Average = 3	160 (38.6%)	173 (44.2%)		
	Low = 4	6 (1.4%)	43 (11.0%)		
	Lowest = 5	1 (0.2%)	13 (3.3%)		
Family financial Status	Very good = 1	3 (0.7%)	3 (0.8%)	136.374	< 0.001*
	Good = 2	47 (11.3%)	29 (7.4%)		
	Average = 3	310 (74.5%)	164 (41.8%)		
	Poor = 4	48 (11.5%)	114 (29.1%)		
	Very poor = 5	8 (1.9%)	82 (20.9%)		
Superstition	No = 0	380 (91.6%)	302 (77.4%)	31.012	< 0.001
	Yes = 1	35 (8.4%)	88 (22.6%)		
Health condition	Very poor = 1	6 (1.4%)	27 (6.9%)	59.668	< 0.001
	Poor = 2	13 (3.1%)	61 (15.6%)		
	Average = 3	79 (19.0%)	78 (19.9%)		
	Good = 4	230 (55.3%)	168 (42.9%)		
	Very good = 5	88 (21.2%)	58 (14.8%)		
Severe Chronic Disease	Yes = 0	57 (13.7%)	138 (35.4%)	51.599	< 0.001
	No = 1	359 (86.3%)	252 (64.6%)		
Mental illness	No = 0	412 (99.0%)	298 (76.4%)	98.234	< 0.001
	Yes = 1	4 (1.0%)	92 (23.6%)		
Family mental Disorder History	No = 0	410 (98.6%)	341 (87.4%)	39.160	< 0.001
	Yes = 1	6 (1.4%)	49 (12.6%)		
Family suicide History	No = 0	401 (96.4%)	304 (77.9%)	62.483	< 0.001
	Yes = 1	15 (3.6%)	86 (22.1%)		
Pesticide stored at Home	No = 0	152 (36.8%)	95 (24.4%)	14.586	< 0.001
	Yes = 1	261 (63.2%)	295 (75.6%)		
Aspiration reached	No = 0	265 (63.7%)	296 (75.5%)	38.051	< 0.001
	Yes = 1	73 (17.5%)	17 (4.3%)		
	NA = 88	9 (2.2%)	4 (1.0%)		
	Don‘t Know = 99	69 (16.6%)	75 (19.1%)		

*Indicated adjusted Chi-square test.

NA indicated not applicable.

In order to investigate the mental health of the subjects, several psychological scales were used. These included the Beck Hopelessness Scale (BHS), the Landerman Social Support Scale (1989), the Dickman Impulsivity Scale (DII), and the State-Trait Anxiety Inventory (STAI). Previous research has indicated that these scales have good reliability and validity in China (Kong et al., 2007; Zhang Y. et al., 2012).

### Data analysis methods

The $X\bar{}\,\text{±}\,S\,D$ was used to describe the quantitative data and *n* (%) was used to describe the qualitative data. Further, *t*-tests, chi-square tests and their adjusted tests methods were used to compare the differences between the two groups. Logistic regression was applied to preliminarily screen the factors. BP neural network and Multilayer Perceptron were used to examine the suicide prediction model. Significance levels were set to *P*≤0.05. The SPSS 21.0 and Matlab R2010b softwares were applied to analyze and establish the model.

### Ethical approval

This study was approved by the ethics committee of Shandong University and written informed consent was obtained from all participants in the study.

## Results

### Demographic description of the sample

The average age of the control group was 25.69 ± 6.16 and the age of case group was 26.84 ± 6.37. Results indicate that there is significant difference in age between two groups (*P* = 0.010). In the control group, there were 202 (48.6%) males and 214 (51.4%) females. In the case group, there were 214 (54.6%) males and 178 (45.4%) females. There was no significant difference by gender between two groups (*P* = 0.086).

Results indicate that there was no significant difference in urban vs. rural residence (*P* = 0.124), but there were significant differences on highest degree earned (*P* < 0.001), years of education (*P* < 0.001), marital status (*P* = 0.020) and whether or not lived alone (*P* = 0.005). The suicide cases were more likely to be older, to have lower levels of education, to be single/never married and to live alone. The statistical description for demographic variables and the comparative results of two groups are shown in Table 1.

### Preliminary screening of predictors of suicide

The prevalence of the possible predictors of suicide for each group is found in Table 2. Comparisons indicated that all items are significantly different between groups. Specifically, the suicide cases are likely to have fewer family members (*P* = 0.003), poor relations with both spouse and parents, lower status in their family, lower family financial status, more superstition, poorer health condition, and higher rates of severe chronic disease, mental illness, family mental disorder history, family history of suicide, pesticide storage at home and unreached aspirations (All *P* < 0.001).

Table 3 describes the measures of mental health for the groups. Results demonstrate that the suicide case group has lower BHS despair scale scores, indicating higher levels of desperation (*P* < 0.001). Further, the suicide cases report lower social support, higher impulsivity and higher anxiety than the control group (*P* < 0.001).

**TABLE 3:** Measures of mental health.

Mental Disorders	Control (*n* = 416)	Case (*n* = 392)	*t’*	*P*
BHS despair scale	73.15 ± 8.01	50.84 ± 13.61	28.130	<0.001^#^
Landerman social support scale	37.14 ± 4.48	29.91 ± 6.07	19.050	<0.001^#^
Dickman impulsivity inventory	14.70 ± 4.94	18.35 ± 6.81	–8.604	<0.001^#^
Spielberger anxiety scale	40.64 ± 6.63	53.33 ± 10.51	–20.324	<0.001^#^

^#^Indicated the adjusted t-test.

### Further screening of predictive factors

In sum, 22 of 24 variables were significantly different by group. In order to create model that is precise and applicable for the general population, several variables were excluded from further analysis. Specifically, highest degree obtained and years of education are highly correlated and lead to collinearity issues. Thus, years of education was excluded. Further, although the spousal relation and relation with parents were both significant different in two groups, spousal relations variable does not apply to everyone. Therefore, a spousal relation was excluded as well. Finally, because the goal is to create a summary and concise model that can be quickly and easily deployed in prevention, however the measures of mental disorder needs numerous and tedious questionnaire, so the mental disorder were also excluded. With these deletions, the final model comprises 16 variables. Although it is possible that excluding any variables may reduce the predictive validity of the model, it is expected that the 16-variable model should have sufficient validity and will have the advantage of being easily implemented by those screening for suicide risk.

### The logistic regression prediction model of suicide

Likelihood Ratio (LR) stepwise Multivariable logistic regression was applied to further screen the predictors of suicide. In the multivariable logistic regression, suicide group or control group (1 = Suicide, 0 = Control) was the dependent variable and the 16 variables were the independent variables. Table 4 shows that there are 10 variables (Age, Highest diploma, Marital status, Relation with parents, Family financial status, Superstition, Severe chronic disease, Mental illness, Family suicide history, Pesticide stored at home) in the optimal multivariable logistic regression, and Nagelkerke *R*^2^ is 0.537. This yields a percentage correct for the control group of 85.3%, percentage correct for the case group of 76.3%, and overall subjects is 80.9%.

**TABLE 4:** The logistic regression prediction model of suicide.

Variables	B	Wald χ^2^	*P*	OR	95% CI for OR
Age	0.045	3.996	0.046	1.046	1.001–1.094
Highest degree	–0.761	27.178	< 0.001	0.467	0.351–0.622
Marital status	–1.031	11.963	0.001	0.357	0.199–0.640
Relation with parents	1.014	46.795	< 0.001	2.757	2.062–3.686
Family financial status	0.669	26.253	< 0.001	1.952	1.511–2.521
Superstition	1.183	18.646	< 0.001	3.264	1.908–5.584
Severe chronic disease	0.666	7.886	0.005	1.947	1.223–3.099
Mental illness	3.346	34.92	< 0.001	28.377	9.355–86.076
Family suicide history	1.777	26.254	< 0.001	5.912	2.996–11.666
Pesticide stored at home	0.488	5.452	0.020	1.629	1.081–2.453
Constant	–4.517	31.472	< 0.001	0.011	—
Nagelkerke *R*^2^	0.537				
Percentage correct of prediction	Control group = 85.3%, Case group = 76.3%, Overall = 80.9%				

Multivariate logistic regression indicates that a diagnosis of mental illness (odd ratio *OR* = 28.377), family suicide history (*OR* = 5.912), superstition (*OR* = 3.264), poor relation with parents (*OR* = 2.757), and severe chronic disease (*OR* = 1.947) are the top five risk factor of suicide in this Chinese sample.

### Back propagation neural network prediction model for suicide

Artificial Neural Network, as an AI technology, imitates the structure and function of biological human brain to establish mathematical models. ANN is an adaptive system (Ma, 2010; Liu and Guo, 2014) consisting of a mass of neuron nodes. ANN has been shown to be an effectively applied in associative memory, non-linear mapping, classified recognition and optimization design (Ma, 2010). BPNN is a multi-layer back-propagation network model trained according to the error reverse propagation algorithm (Liu and Guo, 2014). The current study selected the typical three-layer BP neural network with one input layer, one hidden layer, and one output layer. The 10 variables screened by multiple logistic regression analysis were set as the input variables, and suicide or not (1 = Suicide, 0 = Control) was set as the output variable. The range of neurons number in the hidden layer were determined by $H=\sqrt{M+N}+\alpha$ (Wang and Wang, 1999). (α is the constant from 1 to 10, N is No. of input neurons, M is the No. of output neurons). In this study the range of the hidden layer is 4 ∼13. Hidden layer was set to hyperbolic tangent function and output layer was set to logarithmic sigmoid function.

#### Training of back propagation neural network

**FIGURE 1:**
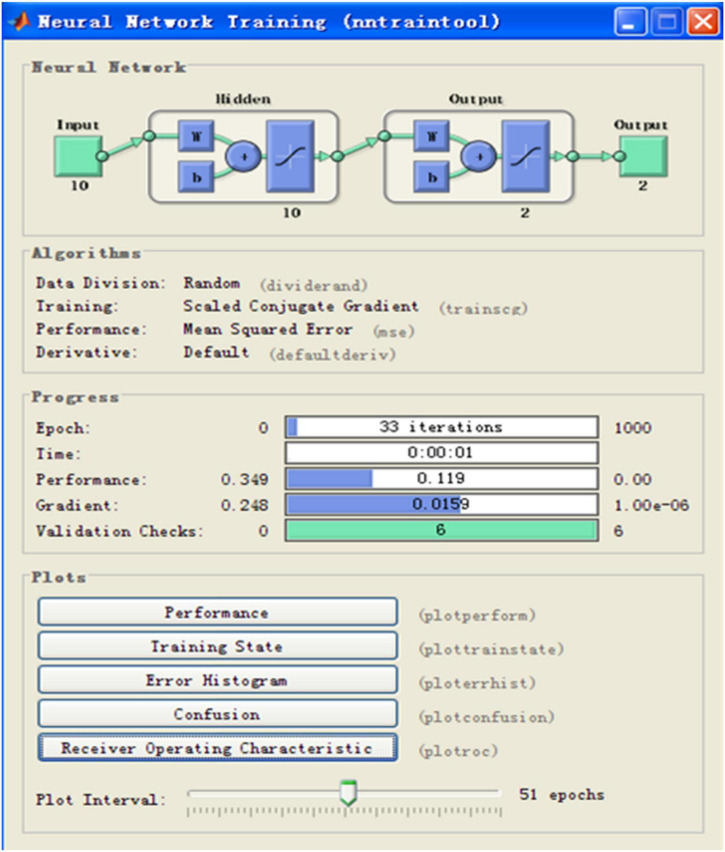
Structure and the parameter settings of the back propagation (BP) neural network.

The Graphical User Interfaces (GUI) of the Matlab R2010b software was used to complete the neural network model. The “Pattern Recognition Tool” module was used to process the classification pattern recognition interface. Data file of X.mat and Y.mat were imported as the input and output neurons. The Matlab R2010b software randomly divided the database into groups for difference testing purposes: 70% as the training sample, 15% as the verification sample and 15% as the test sample. Next, the neural network models were established through the data simulation process repeatedly by Matlab R2010b software under different number of hidden layers. Model evaluation indices, such as sensitivity (Se), specificity (Sp), total coincidence rate (π) and number of iterations are shown in Table 5. It indicates that when there are 10 hidden layers the model evaluation indices are optimized. The structure of BP neural network model, parameter settings and operation results parameters are showed in Figure 1.

**TABLE 5:** Predicted evaluation indices of Back Propagation Neural Network (BPNN) with different hidden layers neurons.

No. of hidden layers	Se	Sp	π	No. of iterations
4	78.1	83.7	80.9	23
5	76.5	88.0	82.4	30
6	75.3	85.5	80.7	29
7	76.3	86.5	81.6	31
8	78.3	86.8	82.7	61
9	79.1	83.9	81.6	36
**10**	**78.1**	**87.5**	**82.9**	**33**
11	79.3	84.9	82.2	37
12	74.0	88.7	81.6	31
13	79.3	85.3	82.4	28
14	77.6	87.0	82.4	36

#### Verification and evaluation of back propagation neural network

**FIGURE 2:**
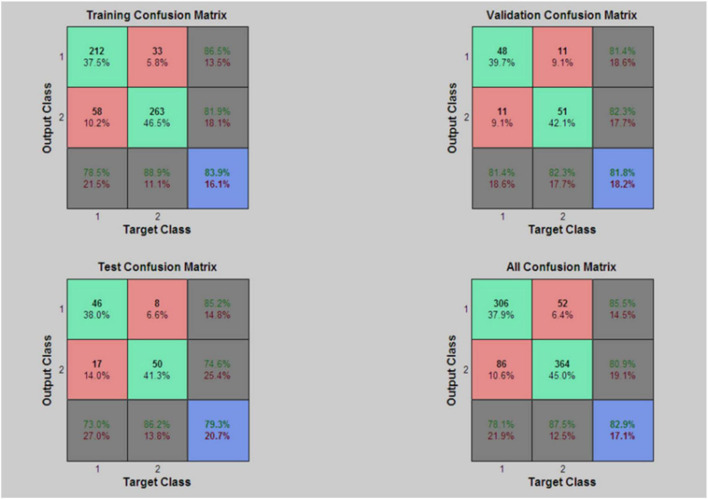
Confusion matrixes of BP neural network model.

**FIGURE 3:**
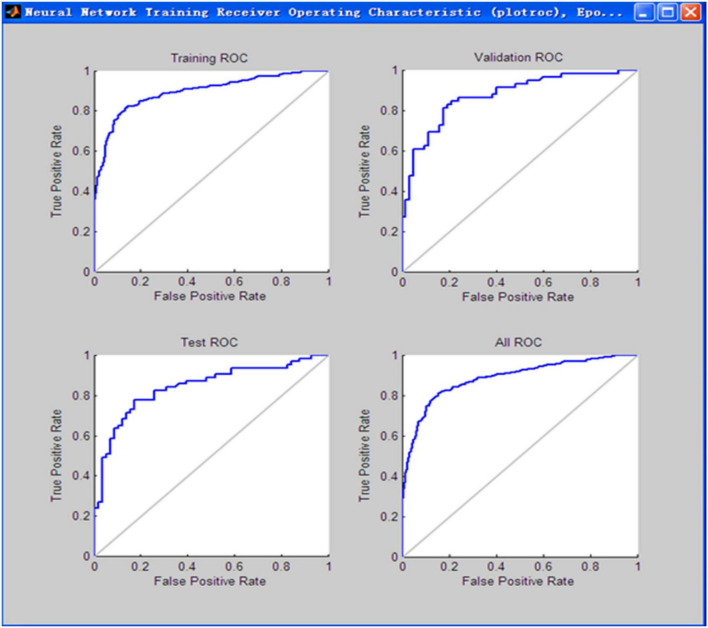
The receiver operating characteristic (ROC) curve of Back Propagation Neural Network (BPNN) for each sample.

The confusion matrixes output by Matlab R2010b are shown in Figure 2. The coincidence rates of each sample are 83.9, 81.8, and 79.3%. The total coincidence rate for all samples is 82.9%. The area under receiver operating characteristic (ROC) curve of each sample is about 82%, which is shown in Figure 3. The confusion matrixes and ROC curve indicate that the discrimination efficiency of the prediction model is superior to the logistic regression model.

### Multilayer perceptron prediction model for suicide

**FIGURE 4:**
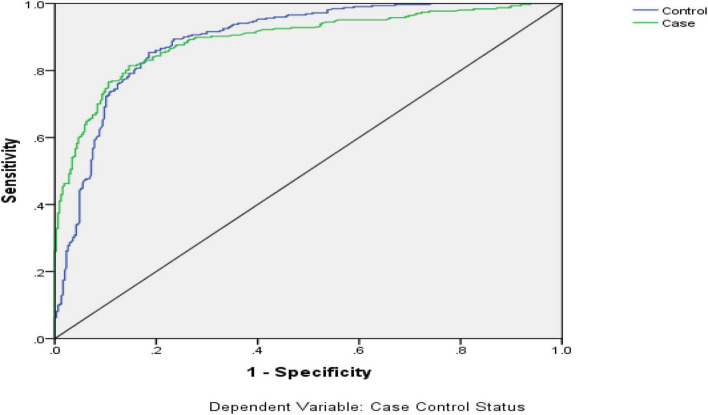
The receiver operating characteristic (ROC) curve of multilayer perceptron prediction model.

**FIGURE 5:**
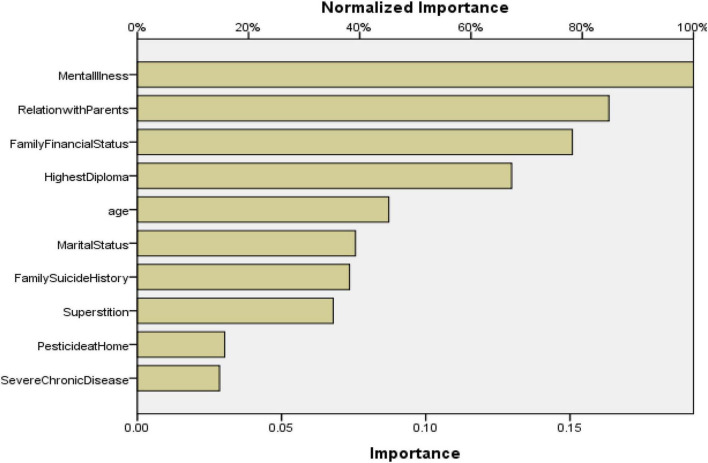
Importance chart of independent variables of multilayer perceptron.

Multilayer Perceptron is another common neural network method, which is widely applied in many fields. The 10 variables identified through multiple logistic regression analysis were set as the input variables and suicide or not was set as the output variable. The SPSS 21.0 software was applied to establish the multilayer perceptron prediction model. In this study the 3-layer (with 1 hidden layer) multilayer perceptron was chosen to establish the prediction model. The database was divided into 3 parts, 70% for the training sample, 15% for the test sample and 15% for the holdout sample. Different architectures and different type activation functions of the hidden layer and the output layer were set to simulate the database repeatedly. The different multilayer perceptron models and the evaluation indices (such as percent correct, area under the ROC curve) are displayed in Table 6. The result indicates that when the activation function of the hidden layer is a hyperbolic tangent, that of the output layer is identity and the number of units in hidden layer is 9 (Model 2), the multilayer perceptron model had the highest AUC. The ROC of Model 2 is displayed in Figure 4. Table 6 and Figure 4 both indicate that the AUC of the optimal multilayer perceptron prediction model is above 90%, which indicates that the discrimination efficiency of the multilayer perceptron model was superior to BPNN model and the logistic regression model. The multilayer perceptron neural network model of suicide has good discrimination efficiency. The importance chart of independent variables is displayed in Figure 5.

**TABLE 6:** The multilayer perceptron models of suicide and the evaluation indices.

Model	Architecture	Activation function		No*.	Percent correct			AUC^#^	
		Hidden layer	Output layer		Train	Test	Holdout	Control	Case
M1	Automatic	Hyperbolic tangent	Softmax	6	78.7%	79.5%	80.3%	0.869	0.869
**M2**	**Custom**	**Hyperbolic tangent**	**Identity**	**9**	**82.9%**	**82.0%**	**80.7%**	**0.902**	**0.902**
M3	Custom	Hyperbolic tangent	Softmax	9	79.7%	75.9%	87.1%	0.872	0. 872
M4	Custom	Hyperbolic tangent	Hyperbolic tangent	9	79.1%	84.8%	78.1%	0.876	0.876
M5	Custom	Hyperbolic tangent	Sigmoid	9	76.0%	83.3%	68.9%	0.855	0.855
M6	Custom	Sigmoid	Identity	9	80.2%	82.4%	76.9%	0.885	0.885
M7	Custom	Sigmoid	Softmax	9	82.1%	84.2%	80.6%	0.884	0.884
M8	Custom	Sigmoid	Hyperbolic tangent	9	81.8%	77.5%	78.9%	0.891	0.891
M9	Custom	Sigmoid	Sigmoid	9	83.2%	78.5%	83.5%	0.883	0.883

*Indicates the number of Units in Hidden Layer.

^#^Indicates area under the receiver operating characteristic (ROC) curve.

## Discussion

The multivariate logistic regression predictor model screened 10 main predictive factors for suicide and overall percentage correct was 80.9%. The total coincidence rate was 82.9% and ROC curve was about 82.0% in BPNN prediction model and the AUC of the optimal multilayer perceptron prediction model was above 90%. The current study verifies the hypnosis that the neural network prediction models have higher accuracy rates than traditional statistical methods. It is advisable to apply traditional statistical methods combined with neural network method.

Although there is considerable previous literature on suicide behavior (Zhang X. et al., 2012; Zhang Y. et al., 2012; Lyu and Zhang, 2014; Oexle and Rusch, 2018; Shah et al., 2018; Tong et al., 2018), the aim of most previous studies was to describe and identify risk factors for suicide. However, much of the prior work on prediction of risk has involved traditional linear statistical models. The current study aimed to establish the different prediction model of suicide and verify the application of the neural network model and compare the accuracy of the discriminant effects.

There are 10 variables identified through multivariate logistic regression, which are the main predictive factors for suicide. The odd ratio (*OR*) values indicate that mental illness (*OR* = 28.377), family suicide history (*OR* = 5.912), superstition (*OR* = 3.264), poor relation with parents (*OR* = 2.757), and severe chronic disease (*OR* = 1.947) are the most important risk factors of suicide in China. The results of current study are in accordance with previous research (Rajalin et al., 2012; Zhang X. et al., 2012; Zhang and Lv, 2013).

Similarly, this study yielded results consistent with prior work in mental health concerns. Greater desperation (Pompili et al., 2011; David Klonsky et al., 2012), lower social support (Rothon and Stansfeld, 2012; Saha et al., 2012; Panayiotou and Karekla, 2013), higher impulsivity (An et al., 2010; Gvion and Apter, 2011; Doihara et al., 2012) and higher anxiety were all significant risk factors for suicide (*P* < 0.001). However, because these variables are not quickly and easily measured they were excluded in the model establishment.

Comparing the three prediction models, the percentage correctly identified of control and case groups is 85.3 and 76.3%, respectively, and the overall percentage correct is 80.9% in the logistic regression model. For the BPNN prediction model, the coincidence rates of each sample were 83.9, 81.8, and 79.3%. The total coincidence rate for all samples was 82.9% and the area under ROC curve was about 82.0%. The coincidence rate is indeed not too high, the reason may be the research data come from the social survey and there are some information biases different to be controlled. Therefore, the conclusion can be drawn that the forecast effect of the BPNN exceeds the logistic regression model. The AUC of the optimal multilayer perceptron prediction model is above 90%, higher than even that of the BPNN model (82.0%). Thus, discrimination efficiency of the multilayer perceptron model was superior to BPNN model and the multilayer perceptron model basing on the neural network is the best prediction model.

The current study verifies that the neural network prediction models have higher accuracy rates than traditional statistical methods. The reason may be the advantages of the ANN. ANN can imitate the mechanisms of cerebral neurons to identify the complex, non-linear relationship of variables and not require specific distribution of the data. The multilayer perceptron model basing on the neural network appears to have clinical and preventive value to distinguish high-risk populations for suicide, and also has important theoretical value for developing auxiliary clinical diagnostic system for psychiatrists. The neural network prediction model based on BPNN and multilayer perceptron can be output and saved, and the saved model can be integrated with network technology to develop website diagnostic systems to identify suicide risk.

Although the neural network method is suitable for identifying potential suicide cases and has greater accuracy than logistic regression method, the neural network method still has strengths and weaknesses. The logistic regression gives the regression coefficients explicitly and is convenient for us to explain. Although the neural network model is not as helpful to identify predictor variables in the preliminary stages of research, it is superior in discriminant efficiency and data simulation. However, the neural network method still has some weaknesses. First, although the structure, weight coefficient and threshold value of neural network model are given, the parameters may change in different data simulation process, making it difficult to make professional interpretations. Second, there are many implicit relationships among variables, so the fixed relationship among them could not be easily displayed. It may be advisable to apply traditional statistical methods combined with neural network models.

## Conclusion

In order to establish brief, accurate, and widely applicable prediction model of suicide, the current study used logistic regression, BPNN and multilayer perceptron methods to establish and compare the accuracy of three prediction models.

The overall percentage correct of samples is 80.9% in logistic regression model. The total coincidence rate for all samples was 82.9% and the area under ROC curve was about 82.0% in the BPNN prediction model. The AUC of the optimal multilayer perceptron prediction model is above 90% in multilayer perceptron model. Although the classification effect is not high excellently, the current study verified the hypothesis that the neural network prediction models have greater accuracy than traditional statistical methods. The discrimination efficiency of the multilayer perceptron model was superior to BPNN model and it is the best prediction model of suicide.

Although the neural network method is suitable to identify suicide cases and is more accurate than the logistic regression method, the neural network method still has strengths and weaknesses. It may be advisable to apply both traditional statistical methods and neural network models.

The neural network prediction model of suicide based on multilayer perceptron and BPNN has clinical and preventive significance to distinguish high-risk populations, and it has important theoretical significance for developing an AI auxiliary clinical diagnostic system for psychiatrists.

## Limitations

Firstly, only the general macroscopical variables were considered, the microcosmic and fussy variables were excluded in this study. Secondly, the processes of data simulation were manually operated by software, and the times and the amount of simulation processes were limited. Further study should try to operate the simulation processes by computer automatically. Thirdly, data collected from different sample may lead to the bias of results. In the end, the effective variables for establishing successful classification still need to be filtrated by the two methods in the future. The more excellent, robust and successful machine learning algorithms should be investigated in the next research.

## Data availability statement

The original data is available upon reasonable request, further inquiries can be directed to the corresponding author.

## Ethics statement

This study was reviewed and approved by the Ethics Committee of Shandong University and written informed consent to participate was obtained from all participants in the study.

## Author contributions

JL contributed to the data analysis, conception, and manuscript writing. HS revised the manuscript and funded the publishing charge. JZ contributed to the data collection and conception. JN polished the manuscript and English language. All authors listed have made a substantial, direct, and intellectual contribution to the work, and approved it for publication.
